# Supplementary material for: Identification and functional analysis of non-coding regulatory small RNA FenSr3 in Bacillus amyloliquefaciens LPB-18
Source: PeerJ. 2023 May 15;11:e15236. doi: 10.7717/peerj.15236 (PMC10194069; doi:10.7717/peerj.15236)
Supplement: Supplemental Information 4 [file peerj-11-15236-s004.zip › KO/CK-vs-T1_map/map04211.html]

KEGG PATHWAY: Longevity regulating pathway - Reference pathway


|  |  |
| --- | --- |
| **Longevity regulating pathway - Reference pathway** |  |

[
Pathway menu
| Organism menu
| Pathway entry
| Show description
| User data mapping
]

Regulation of longevity depends on genetic and environmental factors. Caloric restriction (CR), that is limiting food intake, is recognized in mammals as the best characterized and most reproducible strategy for extending lifespan. Four pathways have been implicated in mediating the CR effect. These are the insulin like growth factor (IGF-1)/insulin signaling pathway, the sirtuin pathway, the adenosine monophosphate (AMP) activated protein kinase (AMPK) pathway and the target of rapamycin (TOR) pathway. The collective response of these pathways to CR is believed to promote cellular fitness and ultimately longevity via activation of autophagy, stress defense mechanisms, and survival pathways while attenuating proinflammatory mediators and cellular growth. Furthermore, there is evidence supporting that life span extension can be achieved with pharmacologic agents that mimic the effects of caloric restriction, such as rapamycin, via mTOR signaling blockade, resveratrol, by activating SIRT1 activity, and metformin, which seems to be a robust stimulator of AMPK activity. As an aging suppressor, Klotho is an important molecule in aging processes and its overexpression results in longevity.

|  |  |  |
| --- | --- | --- |
| Reference pathway | 184% 150% 122% 100% 82% 67% 55% | 图片下载 |
